# A Bandwidth Control Arbitration for SoC Interconnections Performing Applications with Task Dependencies

**DOI:** 10.3390/mi11121063

**Published:** 2020-11-30

**Authors:** Salvador Ibarra-Delgado, Remberto Sandoval-Arechiga, José Ricardo Gómez-Rodríguez, Manuel Ortíz-López, María Brox

**Affiliations:** 1 Center of Research, Innovation and Development in Telecommunications (CIDTE), Academic Unit of Electrical Engineering, Autonomous University of Zacatecas, Zacatecas 98000, Mexico; rsandoval@uaz.edu.mx (R.S.-A.); jrgrodri@uaz.edu.mx (J.R.G.-R.); 2 Department of Electronic and Computer Engineering, University of Cordoba, 14071 Córdoba, Spain or el1orlom@uco.es (M.O.-L.); el1brjim@uco.es (M.B.)

**Keywords:** System-on-Chip, arbiter, interconnection, bandwidth control, quality of service

## Abstract

Current System-on-Chips (SoCs) execute applications with task dependency that compete for shared resources such as buses, memories, and accelerators. In such a structure, the arbitration policy becomes a critical part of the system to guarantee access and bandwidth suitable for the competing applications. Some strategies proposed in the literature to cope with these issues are Round-Robin, Weighted Round-Robin, Lottery, Time Division Access Multiplexing (TDMA), and combinations. However, a fine-grained bandwidth control arbitration policy is missing from the literature. We propose an innovative arbitration policy based on opportunistic access and a supervised utilization of the bus in terms of transmitted flits (transmission units) that settle the access and fine-grained control. In our proposal, every competing element has a budget. Opportunistic access grants the bus to request even if the component has spent all its flits. Supervised debt accounts a record for every transmitted flit when it has no flits to spend. Our proposal applies to interconnection systems such as buses, switches, and routers. The presented approach achieves deadlock-free behavior even with task dependency applications in the scenarios analyzed through cycle-accurate simulation models. The synergy between opportunistic and supervised debt techniques outperforms Lottery, TDMA, and Weighted Round-Robin in terms of bandwidth control in the experimental studies performed.

## 1. Introduction

Embedded applications on Systems on Chip (SoC) present task dependencies and share resources such as memories, peripherals, and accelerators. Real application tasks have data needs that translate into dependencies (expressed as graphs where nodes are the tasks, and the links are the dependencies with data rates as weights) [1]. The system assigns application tasks into one or more processing elements transforming its dependencies to data transactions among processing elements. However, more than one application can run on SoC, which complicates sharing the interconnection and other resources.

The central performance bottleneck of current Systems on Chip resides in the on-chip interconnection rather than in the processing of its elements [2,3,4,5,6]. The electronics industry can presently integrate many processing cores, memories, and IP cores in a single chip, which intensifies this problem. The bottleneck becomes even tighter when the processing elements need to share resources (buses, memory, ports, hardware accelerators, etc.) and cause contentions.

An SoC design must fulfill requirements and constraints imposed by the applications running in the system (such as guaranteeing a delivery time, ensuring specific bandwidth allocation, reducing energy consumption, minimizing area and size, etcetera) [7]. It is then necessary to have control mechanisms in the interconnection system that allow the fair coexistence of the different processing elements in an SoC. In a system composed of various hardware accelerators, the critical factor is the containment of the memory controller [8]. If this controller regulates memory access, the accelerators will meet the Quality of Service (QoS) requirements.

The options for interconnections in the SoC are diverse and depend on the application. Interconnection systems impact directly on the performance of the SoC. The literature then proposed numerous interconnection architectures that increase performance (buses, crossbars, routers, and combinations). However, there is no single solution to the SoC interconnection problem [9,10].

The different interconnection systems are suited to specific applications and scenarios. The bus-based systems are the alternative for SoCs that integrate a small number of processing elements. Benini and Michele in [11] pointed out that buses work well in systems that incorporate four to eight processing elements. On the other hand, crossbars switches become the right choice when the number of processing elements is in the order of tens, as indicated by Ahmed et al. in [4]. Finally, the so-called Network on Chip (NoC) is the commonly used alternative when the number of processing elements is in the order of tens to hundreds or thousands, as Wang et al. declared in [12].

Regardless of the interconnection system used, all the proposed alternatives require an arbitration system that controls shared resources access. In a bus-based interconnection system, the processing elements compete to obtain access to the bus. In crossbars, two or more masters contend for a slave port. In the case of NoC, two or more flows fight for access to a link. The arbitration system and its policy become a fundamental part of the interconnection system within the SoC.

Arbitration systems traditionally focused on achieving fair access, but to regulate the bandwidth allocated is essential too. In collaboration with other components, they can control the utilization of the processing elements to fulfill QoS requirements of the system. A desirable characteristic of an arbitration system is controlling the bandwidth assigned to each of the processing elements [2]. Different SoC companies present very robust alternatives to interconnect processing elements. However, their implementations are more focused on bringing great flexibility than safeguarding the bandwidth solicited. For example, the interconnection systems used by the company Xilinx called SmartConnect [13] use Round-Robin (RR) as the primary arbitration system. While this ensures fair access to shared elements, it does not allow for regulation of their use. Therefore, it is difficult for this configuration to provide the bandwidth required by any hardware accelerator and meet its QoS requirements.

Despite the extensive literature about buses and arbitration, we believe that bandwidth allocation in arbitration systems is not entirely analyzed. Different studies propose solutions to meet QoS requirements for applications running in SoCs such as [14,15,16,17,18,19,20,21,22,23,24]. Most of these works incorporate additional control elements to the arbitration system to regulate the bandwidth and access to resources. However, the effect that arbitration policies have on regulating bandwidth allocation to resources is not fully studied. This paper evaluates the behavior of different arbitration policies commonly used in SoC in terms of their ability to regulate the use of a resource (access, and throughput/bandwidth) and how it impacts the overall performance of the system. Besides, we propose a new arbitration policy (and its architecture) that controls the bandwidth allocation of a shared resource to meet its bandwidth requirements.

This paper makes the following contributions:

- A cycle-accurate framework to model arbitration policies in a bus-based SoC using SystemC.
- Performance analyses of different arbitration policies regarding their possibility of granting and regulating the allocated bandwidth of a bus-based interconnection.
- Derived from the analyses above mentioned; we identify conditions where Weighted Round-Robin policy causes a deadlock in applications with task dependencies.
- A novel deadlock-free arbitration policy with fine-grained bandwidth control based on budgets for every communication transaction.
- Analysis of the architecture of the budget policy implemented in FPGA.

We present the rest of this work as follows: Section 2 shows the related work. Section 3 describes the System Model. Section 4 identifies some issues that have standard arbitration policies in terms of bandwidth control. Section 5 shows our proposal. Section 6 presents the results. The discussion of the results obtained is in Section 7, and finally, Section 8 integrates the conclusions and future work.

## 2. Related Work

This section classifies the related work in two parts: the first one, literature regarding QoS metrics, and the second one, regarding arbitration policies. An effective arbitration policy must have the following features [2]:

- to grant fast high-priority access to the shared resource while avoiding starvation for low priority transactions.
- to enable a delicate control of the communication bandwidth assigned to every processing element.
- to overcome the sensitivity of system performance to various communication patterns generated by the SoC applications.

### 2.1. Quality of Service Metrics

Most studied QoS metrics are access, latency, utilization, Equality of Service (EoS), and bandwidth. Arbiters assign resources with fair, prioritized, or regulated policies, which impact on the metrics above. Every policy or allocation technique has pros and cons affecting applications running on the SoC. Here, we use buses to explain arbitration policies, but switches and routers use them too. Table 1 condenses the most significant related work to our knowledge in terms of arbitration policies. The following is a brief explanation of the aim of each of the metrics used in the classification:

- **Access**.
  Starvation is a result of unfair access arbitration (an entity requesting necessary resources never gets access). The fair access policies overcome the starvation problem guaranteeing an allocation technique where resources are accessible to every request.
- **Latency**.
  Latency is a measure for QoS for real-time applications. Arbitration policies must guarantee application response latency within a specified time constraint.
- **Utilization**.
  This metric focuses on maximizing the utilization of the interconnection within an SoC and increasing the performance of the system to comply with the restrictions established to the applications running within the SoC.
- **EoS**.
  Equality of Service comprises several QoS metrics; it refers to the overall experienced service for every processing element attached to the interconnection system. This metric has a more significant impact on NoCs due to the diverse spatial traffic patterns that affect different parts of the NoC.
- **Bandwidth**.
  As we mentioned above, SoCs need fine-grained bandwidth control. This bandwidth control will permit a bandwidth to every processing element and accurately predict latencies and throughput to applications running in the SoC.

### 2.2. Most Used Arbitration Policies for Bandwidth Control

The second classification indicates the arbitration policy used by the different proposals to reach the QoS metric. Many works studied well-known arbitration policies: Round-Robin, probabilistic access such as Lottery or derivatives, Time Division Access Multiplexing (TDMA), static or dynamic priority access. According to the established scenario, other approaches base their proposal on reconfiguring traditional arbitration policies (Round-Robin, TDMA, Priority, Lottery). Finally, many papers integrate additional monitoring, scheduling, control, etc., to conventional arbitration policies to help meet QoS requirements. In Table 1, we present our classification according to the established metrics. However, below we focused on the more relevant works concerning bandwidth control.

Poletti et al. [2] present a slot reservation technique, a combination of TDMA and Round-Robin policies. A high priority master safeguards bandwidth by periodically assigning a slot. In contrast, a Round-Robin arbiter grants access to the other masters to avoid starvation. The authors present a simple solution that guarantees the bandwidth to one master in the system. We observe that a fine-grained bandwidth control for every master is still missing.

Xu et al. [31] present a work that aims to control dynamically the bandwidth assigned to the processing elements of an SoC with the AMBA-AHB interface. In their work, they propose to modify the Lottery ticket generator; the more waiting time accumulated in the previous contests, the more tickets assigned to a processing element. Although the authors show that their proposal distributes the bandwidth better than other arbitration policies such as Round-Robin or Lottery, the tests they carried out work in a dense traffic situation. The probabilistic arbiters behave best in these scenarios. We notice the absence of experimental studies where bandwidth control corresponds to ticket assignation in the proposed technique. Section 6 in this paper carries out the missing experimentation.

To provide a proportional bandwidth allocation, Li et al. [21] present a dynamically adaptive priority arbitration algorithm. The arbitration policy records the number of requests generated by a particular master and the whole number of requests generated in the system. The weight assigned to a master is (number of master requests)/(number of requests in the system). The arbiter system calculates this value and reassigns it periodically. In their proposal, the authors present a scheme with two levels of priority: the first level adopts the adaptive arbitration algorithm; the second level adopts a fixed priority scheme to prevent starvation.

In this approach, we perceive that there is no regulatory element in the system. The more bandwidth a master demands, the higher weight it gets. Besides, the system suffers from inertia because it works with the values calculated in the previous period. The system cannot know a priori the demand for bandwidth that a master can make in a given period. While the paper indicates that a proper time selection can satisfy practical applications, it is not well suited to applications transmitting data bursts.

Akesson et al. [22,23] present a regulator system based on network calculus called Credit-Controlled Static-Priority that aims to program and regulate access to the shared resources in an SoC. This system tries to avoid over-assignment to masters that generate a large number of requests. The control system consists of a regulator that imposes restrictions on service rate and a static priority planner. The regulator determines which service requests are eligible for scheduling at a particular time. For its operation, the regulator takes into account the number of transactions performed by a master. The transactions assigned to a requester consists of two parameters: the burstiness and service rate. Three restrictions must be met for a configuration to be valid: first, the given service rate must be at least equal to the average application rate; second, it is not possible to assign more transactions than the resource can service in a given time –to keep the system in a stable state and a finite buffer size–; third, the assigned burstiness must be large enough to accommodate a unit of service. We observe that regulation effects in system performance shown are not entirely studied.

In [17], the authors proposed a bus arbitration for shared memories called Priority Division (PD). Their work aims to achieve high bus utilization while ensuring a fixed bandwidth. They accomplish this by combining a fixed-priority arbitration policy with TDMA. Unlike TDMA, a timeslot does not belong to a master; a master has a priority on a timeslot. To guarantee bandwidth to all masters, each master must have the highest priority on at least one timeslot. The master with the highest rank in the timeslot takes it when it requires and only gives it out when it is inactive. Although this work ensures maximum utilization of the bus, we notice that it missed studies of how slot priorities influence the precise bandwidth control for each of the masters in the system.

Yang et al. [15] describe an asynchronous arbiter called Adaptive Priority Round-Robin to provide each master its bandwidth needs. Supporting Round-Robin arbitration, the system has a unit called Priority Module, which assigns each master a weight to fulfill the bandwidth requirements. When a master obtains the bus, its weight value is decreased by one; if this value equals zero, it reaches its bandwidth requirement and no longer enters the contest. Access is only allowed to those competitors who have not met their quota. In terms of bandwidth distribution, this approach behaves relatively well, especially in extreme traffic scenarios mentioned by the authors. However, the paper does not present evidence of how bandwidth distribution relates to system performance.

Slijepcevic et al. [20] report an arbiter that achieves fair bandwidth sharing with different size transactions. The authors propose a Credit-Base Arbitration to ensure fairness among processing elements in terms of the number of clock cycles they hold the bus. To achieve this, they assign a maximum credit that each candidate can have. This credit starts at zero and is increased proportionally in each clock cycle according to the bandwidth ratio that each processing element wishes to obtain. Only when they reached their maximum credit elements can contend. When a processing element gets access to the bus, its credit decreased proportionally.

Although this paper shows a scheme to distribute the bandwidth, we observe that it lacked indications of how the bandwidth spreads among the masters or how this affects the total performance of the system.

Pagani et al. [8] introduce a bandwidth reservation scheme for the AMBA-AXI standard. An AXI Budgeting Unit (ABU) attached to each master in the SoC is responsible for the bandwidth reservation by tracking the number of transactions issued by the master. Also, at a general level, the ABU Controller is in charge of providing and adjusting the ABU budget. When a master spends its quota, he can no longer make transactions until the system reloads its credit. The mechanism presented by the authors, which they call traffic modeler, regulates the rate at which traffic arrives at the arbitration instances in the interconnection system. Although this work essentially contains the traffic of the masters on the AXI bus, there is no evidence of how the regulator affects its overall performance and how much bandwidth has each master.

While all of the above approaches allow for compliance with the bandwidth requirements established for them, they do not specify the contribution of the arbitration policy to bandwidth distribution. There is no evidence of how the arbitration policy influences bandwidth allocation. Here we present an arbitration policy that resolves the fine-grained bandwidth control effectively.

With the arrival of new paradigms for on-Chip interconnection systems such as Networks-on-Chip, the literature opened several research lines in this area. However, most researchers have devoted their efforts to study and propose solutions to the new challenges that Networks-on-Chip present (mapping, switching, routing, etcetera). Typical implementations of channel arbitration for Networks-on-Chip routers commonly use Round-Robin since it is simple, fast, and uses fewer resources. Researchers set their mind to Round-Robin, which provides fair access, avoids starvation, and left the arbitration issues to a second term. Our contribution is to return the attention of the researchers to arbitration issues since there are still gaps in the literature to be filled in this sense. We aim to develop a new arbitration policy that provides fine-grained control of a resource bandwidth’s share that increases SoCs’ performance predictability.

## 3. System Model

**Figure 1:**
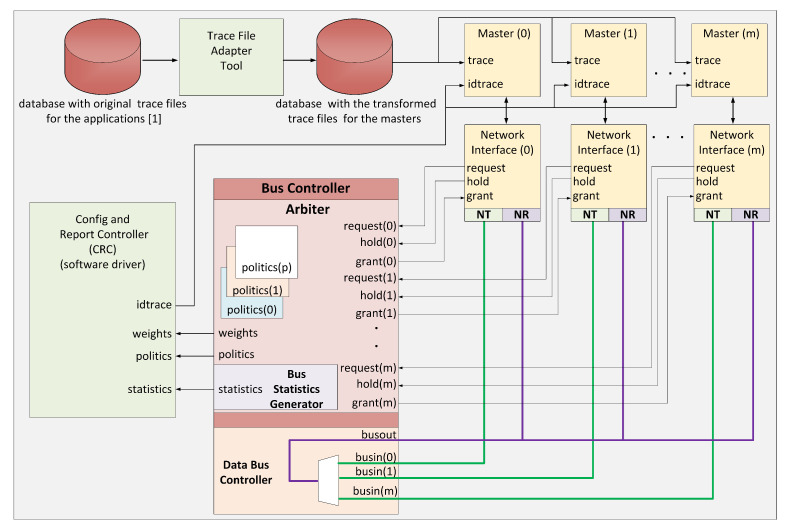
System architecture used for performance evaluations. Databases, Trace File Adapter Tool, and CRC use software implementations.

To evaluate the behavior of arbitration policies in terms of their ability to allow for a differentiated use of a shared resource, we developed in SystemC a system that simulates an SoC with a bus-based interconnection system. The system works cycle-accurate. In Figure 1, we can see the architecture of the evaluation system developed. The system establishes the communication between its elements through a multiplexed bus with 32 bits wide. The main elements of the evaluation system are:

- **Bus Controller** (BC) is the central element of the interconnection system. Three elements constitute it: the first one is the arbiter, who according to the implemented policy, is in charge of granting access to one of the masters who request the media; the second one is the Bus Statistics Generator, which is in charge of keeping the accounting of the bus utilization at a general level and for each master. Also, it registers the execution time for both applications and masters; the third element is the Data Bus Controller in charge of establishing the route so that a master can carry out a transaction.
- **Network Interface** (NI) is in charge of the communication with the other elements of the bus; it is the one that requests the bus to the arbiter and transmits the information when having the bus control. It is in charge of releasing the bus when it is no longer needed.
- **Masters** are responsible for running the SoC applications; they transmit the results of the tasks performed to the interconnection system through the NI they have connected. Each of the masters can execute a set of tasks from a specific application. The configuration unit tells the master which subset to use, and it takes the corresponding trace file from a trace database per master.
- **Config and Report Controller** (CRC), this element has two functions: first, to set configuration parameters with which the Bus Controller will operate; secondly, to collect the operation statistics of the Bus Controller. Table 2 shows the parameters with which the Bus Controller will work. It also shows the statistical variables that are collected.
- **Trace File Adapter Tool** as a result of the mapping process established in [1], each scenario generates a trace file. We take this file with this tool and produce a separate trace file for each master in the application.

To evaluate the performance of the arbitration policies, we established the following assumptions and metrics:

- m(i) is the number of masters running the application(i).
- *n* is the number of applications running in the SoC.
- Master throughput, is the throughput in bits/cycle of the master(j) running the application(i). And is given by:
  (1) $$Th_{master}\left(i,j\right)=\frac{T_{xflits}\left(i,j\right)\cdot flitsize}{T_{masterexec}\left(i,j\right)}$$
- Master bus utilization percentage, is the percentage of bus utilization that the master(j) used when running the application(i). And is given by:
  (2) $$U_{master}\left(i,j\right)=\frac{T_{xflits}\left(i,j\right)}{U_{b}+I_{b}}\cdot 100$$
- Total running time, is the total time it takes the set of applications running in a scenario to complete all the tasks. And is given by:
  (3) $$T_{total}=\max_{i=1}^{n}T_{appexec}\left(i\right)$$
- Application throughput, is the throughput in bits/cycle of the application(i). It is given by:
  (4) $$Th_{app}\left(i\right)=\sum_{j=1}^{m_{i}}Th_{master}\left(i,j\right)$$
- Application bus utilization percentage, is the percentage of bus utilization when running the application(i). And is given by:
  (5) $$U_{app}\left(i\right)=\sum_{j=1}^{m_{i}}U_{master}\left(i,j\right)$$
- Overall throughput, is the total system throughput in bits/cycle when all applications running. It is given by:
  (6) $$Th_{overall}=\sum_{i=1}^{n}Th_{app}\left(i\right)$$
- Overall bus utilization percentage, is the total percentage of bus utilization when all applications running. It is given by
  (7) $$U_{overall}=\sum_{i=1}^{n}U_{app}\left(i\right)$$

**Figure 2:**
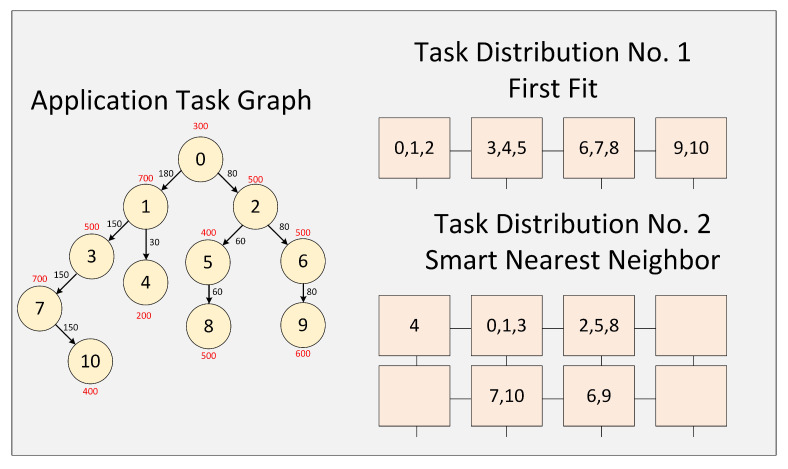
Example of mapping an application using two different techniques [42].

A set of interdependent tasks correspond to the applications that run on our system. A Task Communication Graph (TCG) expresses the tasks and their relationships for every application. A TCG consists of a set of linked nodes (the tasks) and communication links through which the data flow between the tasks. Figure 2 shows the task graph of one application. Each node executes a task at a specific time (the values in red), and when it concludes, it transmits an amount of data (the values in black) to its child nodes. A node can not execute its task until it receives data from the parent nodes. An application begins its execution in the root node (node 0) and finishes when all the nodes leaves have finished operating (nodes 8, 9, 10).

An application can exploit the computing power within an SoC by using a set of processing elements to execute tasks that constitute it. To do this is necessary to use a technique that allows mapping the task graph of an application within the processing elements attached to the interconnection system of the SoC. Figure 2 shows the result of mapping the task network of an application using two different techniques [42].

The distribution made by each technique can significantly affect the traffic generated in the interconnection system. Our study does not delve into the mapping techniques for a bus-based interconnection system. We use the trace files generated by the suite presented in [1]; in this paper, the authors use a mapping focused on load balancing. This suite does not produce specific traffic patterns for bus-based interconnection systems. However, we can use this mapping because, in our case, the effect we want to measure is the ability to control access to the bus when interdependent tasks use it.

In [1], the authors generated the traffic patterns after the mapping and scheduling process and performance evaluation process. Each application for each topology generates a trace file. This file contains all the information regarding the topology, number of processors, number of tasks, number of links, execution times, message sizes, and interdependencies. We take the trace file generated by the suite and divide it into *m* files according to the number of processing elements involved in the mapping. Each of our files contains the information of the tasks to execute: initial tasks, final tasks, time of execution of the tasks, size of the messages generated by each task, dependencies of each task, and children nodes of each task. When starting the simulation process, each master obtains from the CRC an $ID_{appmaster}$ that tells it which trace file to load and execute.

## 4. Bandwidth Control Problems with Common Arbitration Policies

As stated above, one of the objectives of this study is to understand the degree to which an arbitration policy can regulate the bandwidth allocations in the interconnection of an SoC. This section exposes some drawbacks in the arbitration policies used in the literature to implement a fine-grained bandwidth control.

When in an SoC, there are one or several applications with dependent tasks running, and also, it is necessary to make a differentiated use of resources, traditional policies have limitations. Below are some facts that cause these limitations.

- Round-Robin (RR), is a policy that does not have a control mechanism to allow differentiated bus utilization or bandwidth allocation. Round-Robin focuses its efforts on allowing fair access to the bus.
- Lottery (LTY) [41] is a policy with a probabilistic approach that complicates the ticket allocation computation that ensures the bus bandwidth distribution in proportion to the assigned tickets. We can approximate this computation with a stochastic characterization of the task dependencies, packet size, packet generation times, and so forth.
- Time Division Multiple Access (TDMA), this policy allows the differentiated use of the resources but generally at the cost of decreasing overall performance with coarse-grained bandwidth control.
- Weighted Round-Robin (WRR) [6], in scenarios with dependent tasks, with this policy, there is the possibility of deadlock. A task cannot start its execution until it has received all the messages from its predecessors. For example, an application mapped in two masters. Assuming that the $task_{x}$ in the $master_{0}$ to run needs the message of the $task_{y}$ that is in the $master_{1}$. If the $master_{1}$ has consumed its associated weight, it cannot send the message because the arbiter does not grant it access. On the other hand, the $master_{0}$ is unable to continue working because it has not received the $task_{y}$ message, which causes the application to freeze.
- Weighted Round-Robin Modified (WRRM) [6], this policy allows the bus to a master who has exhausted his quota as long as no master who still has weight is requesting it. This policy aims to increase the utilization of the bus. However, by doing so, the most demanding masters are overcompensated, which prevents them from making an assignment following the established weights.

Given the gap described above, we present Supervised Debt Opportunistic (SuDO), a new arbitration policy that allows contenders for a shared resource to make a differentiated use of it. Our proposal achieves fine-grained bandwidth control avoiding reduction in the overall performance of the system.

## 5. Supervised Debt Opportunistic Arbitration: A Novel Policy

Our policy achieves fine-grain control of the bandwidth—or utilization—assigned to every master in the system. It utilizes counters to record every clock cycle used for every master in the system. The policy manages an accounting balance of the bandwidth assigned. It controls the bandwidth manipulating the value assigned to the counter at the beginning of the process. We describe the arbitration method here.

We start setting a—different or equal—budget for every master in terms of the number of flits allowed to transmit as its share of bandwidth. In a contention round, the masters with the largest number of flits enter to compete in a Round-Robin policy. If only one master has the highest budget, it gets the bus. For every flit transmitted or clock cycle that the master uses, we decrement its value by one unit until it finishes its transaction or runs out of flits. A debt record starts and increments for every flit used until it completes its transaction. If a master runs out of flits or it has a debt, and if no other master requests the bus, it acquires it. However, the arbiter policy records the flits used in this event as a debt. Once every master runs out of flits, they get the budget again. When the master obtains more flits, we subtract the debt, and the result remains as flits to spend. With this mechanism, we ensure that the mean bandwidth assigned stays the same over an observation window.

Our policy has two main features, debt supervision and an opportunistic bus allocation. Debt supervision refers to the accounting of the debt every time we reassigned the budget. Opportunistic behavior refers to the resource allocation to a master that requests the bus when no other masters do, even when the master has no flits to spend.

We use the number of transmitted flits for each master as the metric for utilization or bandwidth. We translate clock cycles to flits defining a flit size as the bus data width. This assumption may change depending on the architecture because a flit transmission may take one or more clock cycles. In Networks on Chip, the one flit equals one clock cycle assumption is commonly valid. We can then transform the flits per clock cycle to bits per clock cycle to have a metric associated with bandwidth allocation.

### 5.1. Architecture

**Figure 3:**
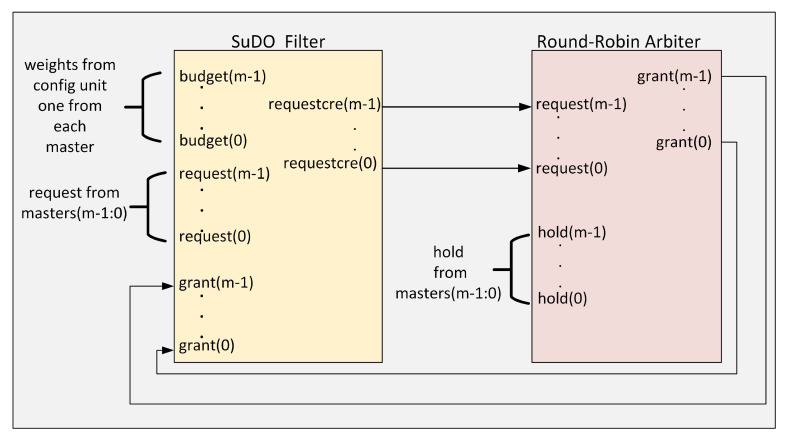
SuDO Arbiter architecture.

Figure 3 shows the architecture of the proposed SuDO arbiter. The architecture consists of two elements: The SuDO filter and a Round-Robin arbiter. The function of the SuDO filter is to process the request signals coming from the masters so that at the output, only those whose number of flits corresponds to the highest budget detected among the competing elements are active. The Round-Robin arbiter resolves ties coming from the SuDO filter, which happens when the account of two or more masters corresponds to the highest budget detected.

For the SuDO filter to operate, it is necessary to provide the budget each master has. The source of these values can come from different sources: a simple set of registers or a processor that allows the dynamic control of these weights under a Software-Defined Network-on-Chip philosophy as in the proposal made by Sandoval-Arechiga et al. in [43]. The number of flits assigned to a master corresponds to the number of clock cycles of the bus. This amount is what enables a differentiated use of the bus.

**Figure 4:**
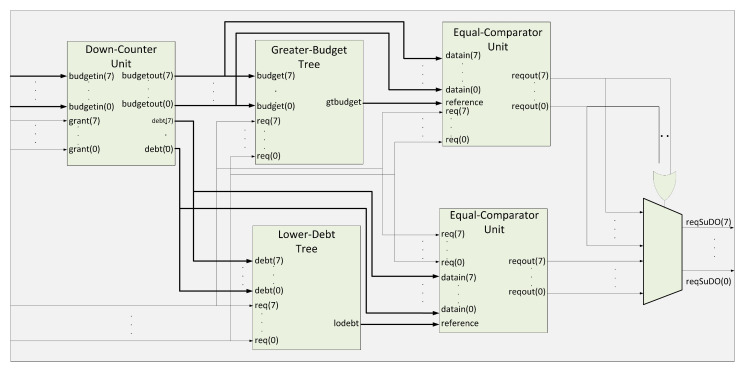
SuDO filter architecture for eight masters.

Figure 4 describes the internal architecture of the SuDO filter. We show an architecture for eight masters because it is a typical size in commercial developments. However, we can extend this architecture up to 32 masters; we present the eight master version for simplicity and explanation purposes. The **Down-Counter** Unit accounts for the balance of the budgets and debts for every master in the system. We use two branches to find the highest and lowest values in the system. The superior tree, **Greater-Budget**, finds the highest amount for flits to spend among the budget of the masters. The inferior tree, **Lower-Debt**, selects the master with the lowest accumulated debt. The **Equal-Comparator** Units choose the requests from the master that corresponds to the input value to compare. The multiplexor selects the requests from one of the branches. If we have at least one master with zero debt, the OR gate sets the upper input to the multiplexor.

**Figure 5:**
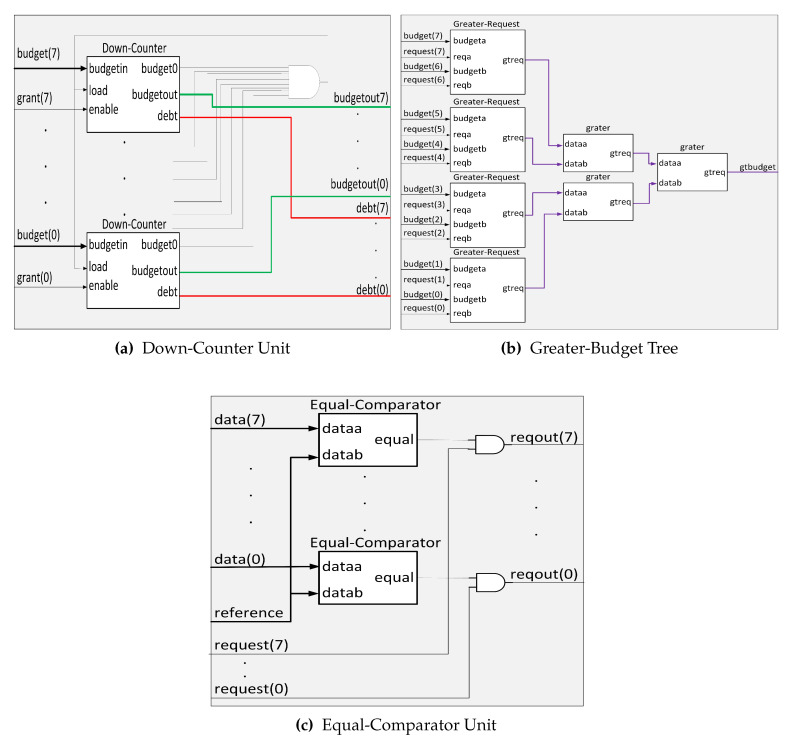
Main SuDO filter units for eight masters.

The **Down-Counter** Unit consists of *m* elements of the **Down-Counter** type one for each master in the system. Figure 5a shows the case for eight masters. The budget of the masters originally specified sets the initial value for each **Down-Counter**. This number will decrease by one unit each clock cycle that the enable signal is active. The enable signal is activated by the grant signal from the RR, indicating that the master associated with this signal has control of the bus. Every **Down-Counter** accounts for the budget and debt for a master, and it has three outputs:

1. *budgetout*, which indicates the number of flits that a master has to spend at a given time.
2. *budget0*, this output means when the internal counter of the **Down-Counter** is equal to zero; this indicates that the master has consumed all its flits.
3. *debt* signal specifies the number of debt flits.

When the *budget0* signal from all **Down-Counters** is active, the *load* signal is activated, and all **Down-Counters** recharge their flits again. An external configuration unit manages this budget value. Moreover, at each recharge, the **CRC** Unit could modify the number of flits. When the internal counter of a **Down-Counter** reaches zero but still requires the bus because the associated master is transmitting a packet, the *budget0* signal is activated. The budget counter value stays at zero. A debt record starts, and it allows the master to conclude with the current transaction.

**Figure 6:**
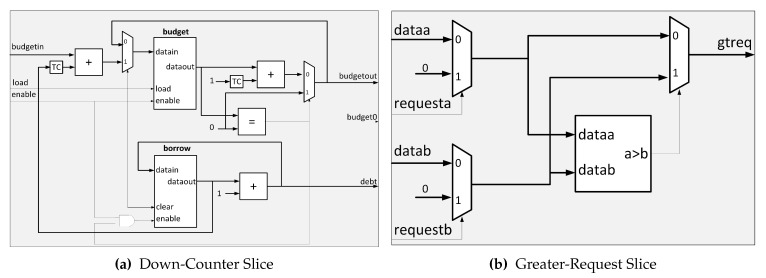
Slices of Down-Counter and Greater-Budget Units.

A **Down-Counter** cell has two registers to record the budget left (superior block) and accumulated debt (inferior block), respectively, as Figure 6a shows. The **TC** blocks are the Two’s complement functions to implement subtraction. The left subtraction block accounts for the budget initialization process to balance the accumulated debt. Every clock cycle that *enable/grant* signal is high; the subtraction block at the right of Figure 6a decrements the budget. Finally, the lower register behaves as an accumulator when the *budget0* signal is high.

SuDO filter uses two comparator trees: **Greater-Budget** to get the maximum budget value, and **Lower-Debt** to obtain the minimum debt value. They differ in the comparison operators, but internally they have the same structure. Here we explain the **Greater-Budget** tree’s architecture but can be easily translated to the **Lower-Debt** changing the > operator for < and *budget(m)* for *debt(m)* signals. The **Greater-Budget** has its first level with m/2 comparators called **Greater-Request**, which present the greater of the two entries at the output. In this comparator, we add the request inputs to indicate whether the next stage of comparators use this value or not. If the signal is high, the data goes into the comparator; otherwise, the value zero. This action makes sense because comparators should only consider the budget of the masters applying for access. We present a cell of this type of element in Figure 6b. In the next tree levels, we use traditional comparators to obtain the highest budget of those masters at the end of the last stage.

The value of the highest budget obtained is compared individually with the budget of each master. A set of comparators named **Equal-Comparator** perform the comparison. Figure 5c shows their architecture. **Equal-Comparator** generates the output *equal=1* if the two values are equal. Finally, the module applies an and operation between the output of these comparators and the original request signal from the associated master. A positive outcome of this operation indicates that the master requests the bus and has the most budget. All outputs are directed to the RR to resolve a tie if one exists.

### 5.2. SuDO Policy Considerations

For its operation, the SuDO policy takes into account the following considerations:

- All masters are assigned a starting budget in terms of flits.
- In an arbitration round, the winning master is the one with the most flits.
- If two or more masters have the same amount of flits to spend that corresponds to the highest number of flits detected in the system, the Round-Robin policy resolves the tie.
- A master who has obtained access to the bus decreases one unit for each transmitted flit.
- A master who runs out of flits will be allowed to complete the current transaction, but a debt record starts.
- A master gets the bus if it requires it even if it has used up his budget, as long as there is no other master with flits to spend is requesting it.
- If two or more masters who have used up their budget apply simultaneously for the bus, the one with less debt gets it.
- If the accumulated debt is the same for all, Round-Robin breaks the tie.
- If a master who has no budget takes the bus, this master accumulates debts in terms of the number of flits used by the bus.
- The arbitration policy reloads the original budget when all masters’ accounts reached zero.
- The policy subtracts the assigned budget and the accumulated debt; the remaining are the flits to spend.

## 6. Results

The characteristics of the applications running on the SoC condition the interconnection performance. To establish scenarios that allow us to make an objective evaluation, we identified features that enable us to classify the applications.

According to how the applications running in the processing elements of an SoC, we identify two types of applications:

1. Application with independent tasks: in this type of application, each of the processing elements executes tasks whose operation does not depend on the execution of another task. Once the processing elements transmit a packet with the task result, they can continue executing pending tasks. There is no need for a response. In this way, they have the possibility of requesting access to the bus again when they complete the execution of their new task.
2. Application with dependent tasks: the applications have tasks that communicate with each other to execute an algorithm. The tasks are distributed in different processing elements to take advantage of their ability to work in parallel and obtain better performance in the processing of the application. The tasks communicate with each other through the interconnection system. A task only can run if it has received all the data from the anterior tasks on which it depends.

According to the type of traffic generated by the applications running on the processing elements of an SoC. We classify the traffic as:

1. Homogeneous traffic: All the masters generate transactions with a similar size payload.
2. Heterogeneous traffic: The size of the packets transmitted by each of the masters varies significantly between them.

The size of the packets transmitted through the interconnection system depends on the application running. Taking as a reference to the traffic patterns presented in [1], we have identified three types of packets:

1. Small-sized packets: with a payload flits in the order of the units. As an example, the mapped FFT-1024 complex algorithm generates packets between five and seven flits payload.
2. Medium-sized packets: which have a payload in the order of tens of flits. Such as those generated by the mapped FPPPP algorithm.
3. Large-sized packets – We observe this size in the mapping of the H264 video encoding algorithm. The generated packets have a payload of between two and three hundred flits.

Our system implemented the RR, LTY, TDMA, WRR, WRRM, and SuDO arbitration policies in the arbiter unit. In a simulation, only runs the police that CRC indicates. Depending on the arbitration policy selected, the CRC associates to each master a weight. We show the policy origin and the purpose of the associated weights in Table 3.

Today, it is common for SoCs to run different task-dependent applications while meeting QoS requirements simultaneously. Wich implies that the arbitration system must provide two capabilities: one, to operate, the ability to avoid starvation and deadlock; two, to achieve QoS, the ability to regulate the utilization of the bus, allowing each processing element a differentiated use of it.

As far as we know, the study of the behavior of arbitration policies in scenarios involving task-dependent applications has been little studied [2]. From our perspective, it is necessary to evaluate the behavior of arbitration policies regarding their capacity to regulate resource utilization. In our case, the bus-based interconnection system. Since controlling the utilization of a resource will depend on the ability to meet the QoS requirements established for the applications.

In the tests, we use two traffic patterns obtained from the suite presented in [1]. The first pattern generates the traffic for the FFT-1024_complex application; this pattern has a high amount of tasks (16,384) with (25,600) communication links; the size of the messages that generate the tasks are in the order of the units (between 5 and 7 flits). The second pattern produced traffic for the FPPPP application; it has a small number of tasks (334) with (1145) communication links; the size of the messages that generate the tasks is in the order of tens (between 50 and 60 flits). Both patterns perform a process of 20 iterations.

We selected these patterns because the FFT-1024_complex application generates intense communication with small packets, while the FPPPP application generates moderate communication with medium-sized packets. Also, these patterns were selected over the other patterns in the suite because, in the tests we performed, they are the ones that generate the highest volume of traffic. A mapping process was carried out on eight processing elements for both patterns because, in our tests with this mapping, we have the best processor/performance ratio. When we run the standalone mode applications, FFT-1024_complex mapping on eight processors achieved a 29.13 bits/cycle performance, and FPPPP mapping on eight processors achieved a performance of 15.95 bits/cycle. To perform the evaluation, we use the previously established metrics: $T_{total}$, $Th_{app(i)}$, $Th_{overall}$, $U_{app(i)}$, and $U_{overall}$.

**Figure 7:**
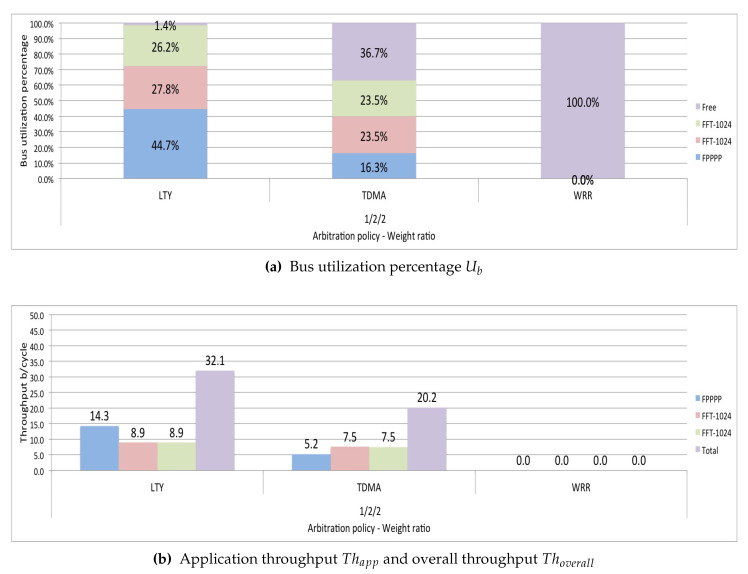
Some problems detected on traditional politics running in a scenario heterogeneous dependent applications. LTY has a deficient bandwidth control. TDMA degrades bus utilization. WRR generates starvation and deadlock issues.

Section 4 indicates the difficulty that some traditional arbitration policies have to control the utilization of the bus, especially when running heterogeneous task-dependent applications. The following are the results of simulating LTY, TDMA, and WRR arbitration policies in a scenario running one FPPPP application and two FFT-1024_complex applications with a 1/2/2 weight ratio. Figure 7 shows that the WRR policy does not generate results because it reaches a deadlock state after running for a period. Figure 7a shows that the TDMA arbitration policy performs a bus distribution that tends to obtain the established weight ratio. However, it translates into an extended idle bus (36.7%). This cost significantly impacts the overall throughput of the system $Th_{overall}$, as shown in Figure 7b. The lottery policy cannot control the utilization of the bus to obtain the ratio established in the weights; in Figure 7a, we can see that the highest percentage of utilization is in FPPPP, which is the application that generates larger transactions. The simulation shows that the two FFT-1024_complex applications had greater access to the bus because they had a more significant number of tickets, but this does not reflect greater bus utilization. In all the tests we conducted with heterogeneous application-dependent scenarios, the behavior of these policies is similar to that shown in Figure 7. In the following tests, to present more concretes results, we eliminated these policies.

Next, we propose a couple of scenarios running applications with dependent tasks. It is of our interest to know to what extent an arbitration policy allows a differentiated resource utilization. We are also interested in observing how this differentiated utilization impacts the overall performance of the system and application execution time.

### 6.1. Scenario 1: Masters Running Different Task-Dependent Applications

In the first test, the proposed scenario runs three applications with dependent tasks, one application is FPPPP, and the other two applications are FFT-1024_complex. The applications run using the RR, WRRM, and SuDO arbitration policies. For WRRM, the weight ratio is 1/1/1; for SuDO, the budget has a weight ratio of 1/1/1. This ratio assumes that the processors of three applications have the same possibility of access to the bus.

**Figure 8:**
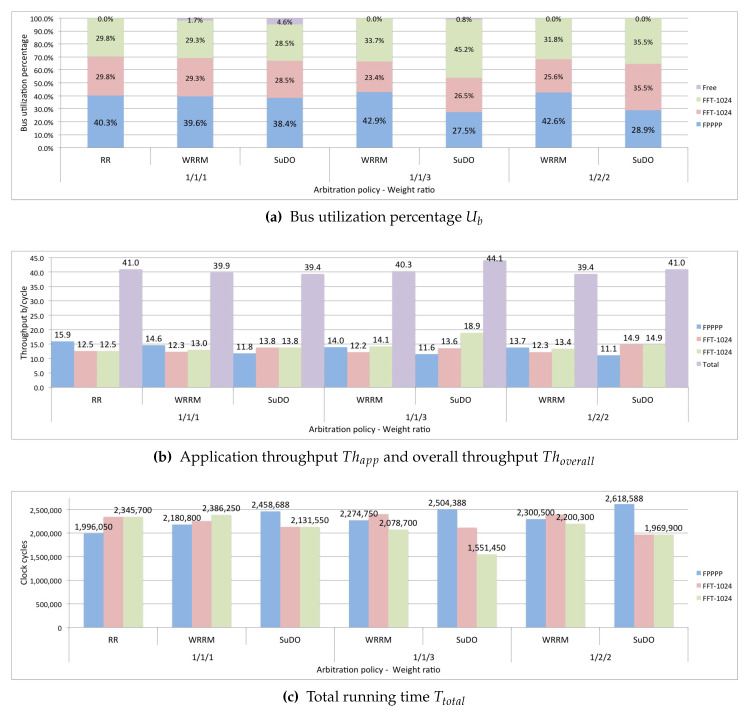
SuDO outperforms other arbitration policies regarding bandwidth control in systems with masters running different task-dependent applications.

Figure 8a shows that the three policies distribute bus utilization relatively equally. For the three arbitration policies tested, the application that obtains the most considerable portion of the bus utilization $U_{app}$ is FPPPP, since the FPPPP application is the one that generates the largest transactions. The overall performance $Th_{overall}$, is similar to all three policies, as seen in Figure 8b being slightly higher for Round-Robin. As for the total running time $T_{total}$, RR presents a slightly running time, as shown in Figure 8c.

In the next test, only run the WRRM and SuDO arbitration policies. In this test, we want to see if an application can make a differentiated utilization of the bus. We set the weighting ratio at 1/1/3, which would mean a theoretically expected bus utilization of 20% for the FPPPP application, 20% for one of the FFT-1024_complex applications, and 60% for the other FFT-1024_complex application. It can be seen in Figure 8a that WRRM cannot correctly regulate bus utilization. Although the FFT-1024_complex application with the highest weight ratio has increased its bus utilization $U_{app}$, this is very far from the desired ratio. We can see that the percentage of utilization gained is at the expense of the other FFT-1024_complex application. SuDO presents a distribution more following the established ratio of weights; However, it is still far from the desired proportion. We can see that the gain is at the expense of the FPPPP application. In terms of performance, we show in Figure 8b that the use of the SuDO arbiter does not affect the overall performance of the system $Th_{overall}$, achieving SuDO get the highest one. As for the total running time $T_{total}$, WRRM presents the shortest time, as shown in Figure 8c.

The third test performed in this scenario sets the weight ratio to 1/2/2. We expect a bus utilization percentage of 20% for the FPPPP application and 40% for each FFT-1024 complex application. The results show that the best distribution of bus utilization $T_{total}$, according to the established relationship, is achieved with the SuDO arbitration policy, as shown in Figure 8a. The overall performance, $Th_{overall}$, is slightly higher for SuDO, as seen in Figure 8b. However, the total running time $T_{total}$ is higher in SuDO than in WRRM, as shown in Figure 8c.

The set of tests that we performed shows that the policy that makes the best control of the bus utilization and the bandwidth is SuDO.

### 6.2. Scenario 2: Masters Running Equal Applications with Dependent Tasks

Another common scenario in SoCs is having different instances of the same application running simultaneously. In this case, we use three instances of the FFT-1024_complex traffic pattern for eight processors. The selection of this application is because the tests we have carried out show that the performance of the system is more compromised when small packets travel in the interconnection system. We run the tests for the same arbitration policies outlined in the previous scenario.

For this scenario, the first test takes as arbitration policies Round Robin, WRRM, and SuDO. The established weight/budget ratio is 1/1/1.

**Figure 9:**
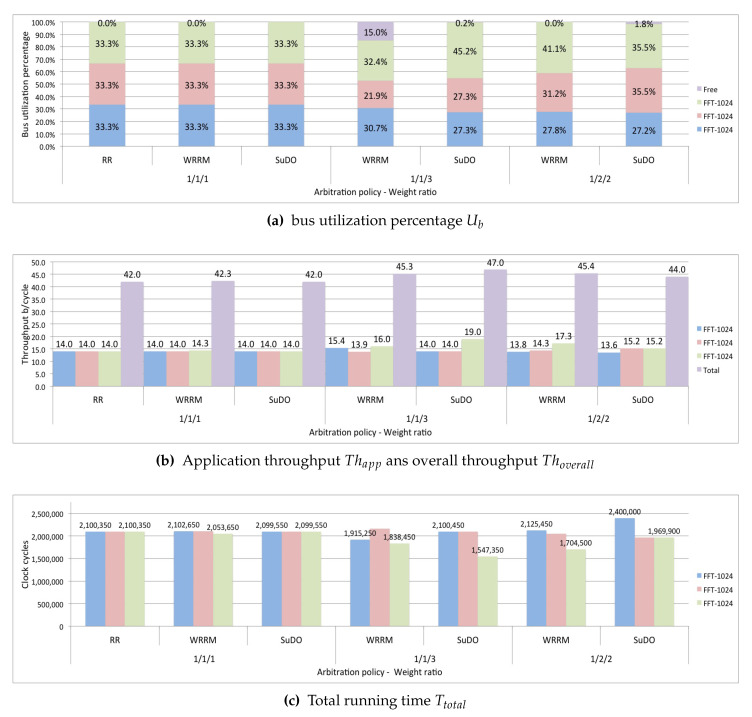
SuDO outperforms other arbitration policies regarding bandwidth control in systems with masters running equal task-dependent applications.

The Figure 9a shows that regardless of the arbitration policy used, each of the three applications obtains the same bus utilization $U_{app}$. The overall performance $Th_{overall}$ and the total execution time $T_{total}$ is practically the same in all three policies, as shown in Figure 9b,c.

The second test in this scenario runs with the WRRM and SuDO arbitration policies. We set the weighting ratio at 1/1/3. Figure 9a shows that WRRM cannot properly regulate bus utilization $U_{app}$; we can see a considerable percentage of idle bus time. SuDO presents a better distribution of bus utilization according to the established ratio. Applications that have the same ratio get the same percentage of bus utilization. The overall system performance $Th_{overall}$ is slightly higher in SuDO, as shown in Figure 9b. Figure 9c shows that WRRM has a lower total running time $T_{total}$.

The last test establishes a 1/2/2 weight ratio, which means that one of the FFT-1024 complex applications has 20% of the bus utilization percentage while the other two FFT-1024_complex applications have 40% each. Figure 9a shows that the SuDO arbitration policy better distributes the bus utilization $U_{app}$. WRRM presents a slightly higher overall performance, $Th_{overall}$, than SuDO, as shown in Figure 9b. The total running time $T_{total}$ is lower in WRRM, approximately 11%, as shown in Figure 9c.

The tests conducted in this scenario show that the policy that makes the best control of the utilization of the bus is SuDO.

Table 4 summarizes the results obtained in the tests carried out in this work.

The tests we have performed show that more than the number of processors in the system, the arbitration policy’s behavior is more influenced by the packet injection rate of each application. For example, in scenarios with task-dependent applications, when we mapped an application in a few processors, the packet exchange is more intensive. In this case, the results are similar to the scenarios tested in this work. When there is a scenario whose applications are not bus-intensive, there is less contention, so the behavior of any arbiter cannot be easily observed no matter how many processors the applications are mapped (few or many).

We test different applications with different transaction sizes. But we observed that FPPPP and FFT-1024_complex are the ones that stress out the system higher. Other applications utilize less the bus; then, we fail to perceive the bandwidth control. Our policy assigns bandwidth to a master, but the arbiter transferred the rest to other masters that needed it if it uses less. In other words, our arbitration policy limits the maximum bandwidth assigned to a master, but the master can use less in a given period. Therefore, to observe more precise bandwidth control, the paper’s scenarios comprise various applications with different transaction sizes that increase bus utilization. Although we used only three applications, they employed eight masters each. Therefore, we use a total of 24 masters in the test. We consider that these scenarios are enough to get reliable conclusions as the bus utilization is adequate to express the possible issues associated with bandwidth control.

### 6.3. Hardware Implementation Evaluation

We implement the hardware architecture of the arbitration policies in the Verilog Hardware Description Language. For the synthesis process, Xilinx’s SoC, Zynq 7020, was used as a reference. The number of masters that are supported by the arbitrators is eight. Table 5 shows the results of the resources used, maximum operating frequency, and energy consumed. The implementation that uses the least amount of resources is Round-Robin, 32 slice registers, and 66 LUTs. WRRM policy consumes 280 slice registers. Most of these in the registers that serve to keep track of the support weights. In the SuDO arbiter case, the number of slice registers increases to 360 because, in this policy, there is a need to keep track of the time debt that a master has. In unit terms, a cell of the RR type consumes three slice registers. A section of WRRM consumes thirty, while a cell SuDO consumes forty slice registers. As for the maximum operating frequency, Round-Robin gets the highest maximum operating frequency. SuDO has the poorest maximum operating frequency; the leading cause is the comparator trees that SuDO uses to find the element with the highest budget or lower debt. These trees make the propagation time high, which considerably decreases the maximum operating frequency. In terms of total power and dynamic power consumption, Round-Robin has a lower consumption. WRRM and SuDO have practically the same consumption characteristics.

The implementation of the proposed policy obtains an excellent compromise between complexity and performance. Table 5 shows the resources used by [44], which offers a different solution -a traffic regulator for every master- for the problem of bandwidth control. It is important to note that the resources shown in Table 5 for [44] are required by each master, which increases the resources used for solutions with multiple masters or cores. Every master added increases one fold the digital logic needed. In contrast, our solution employs fewer resources as we only modify the arbiter in the bus controller. We established that the price paid compared to traditional policies is moderately high; however, compared with the state-of-the-art solutions for bandwidth control, the proposed implementation is less resource-intensive, as Table 5 shows.

## 7. Discussion

Tests conducted on scenarios with task-dependent applications show that SuDO is the policy that allows better control of the bus. When we set the support values to 1/1/1, the behavior of the three tested arbitration policies is very similar in the two scenarios.

When there is a ratio with different weights, WRRM can not maintain this ratio. This behavior is because WRRM allows a processing element to use the bus even though it has already consumed the quota, making this element more resource-intensive. Suppose a processing element frequently requests the bus. In that case, it gets it, in the interest of better overall system performance, but the policy has no control over how much extra bus utilization occurred. This behavior prevents having a fine-grained control of bus utilization. In both scenarios, we can see that the gain in bus utilization that has the application with a higher weight relationship does so at the expense of only one of the other applications. This issue is a consequence of giving the bus to any processing element that requests it even if it has consumed its budget to obtain a higher bus utilization. As shown in scenario 2, the 1/1/3 WRRM ratio has an idle bus percentage of 15%.

In the case of SuDO, the results show that although it does not achieve the desired weight ratio, there is a tendency to achieve it. The main reason why this happens is that applications do not request the bus at a fixed weight ratio because the mapping process tried that the most significant task dependency is between tasks within the same processing element. Given the above, a processing element during specific periods does not request access to the bus. We can use the packet injection rate as a measure to adjust the budget values.

Regarding the overall performance, we can see that taking control of the bus utilization does not significantly impact the arbitration policies evaluated in the scenarios proposed. SuDO, in most of the tests with differentiated weights, presents a slightly higher performance. The price to be paid for achieving a better distribution of bus utilization is the total running time of the applications. In this sense, SuDO in all tests presents a higher execution time of the task that takes longer to execute, approximately 10%.

**Figure 10:**
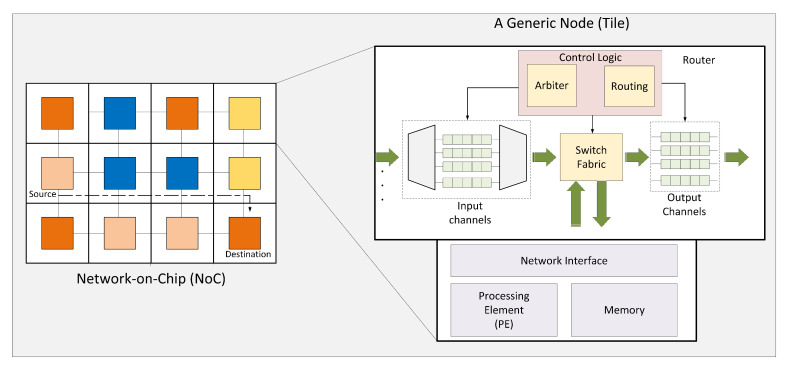
A typical Network-on-Chip architecture. A 3 × 3 2D Mesh topology with its internal tile architecture. Arbitration logic controls the switch fabric and input channels showed in the router’s microarchitecture [45].

We can implement our SuDO policy in other interconnection systems such as crossbar switches and routers. Here we explain such extension in routers, but switches benefit from SuDO policy in the same manner. Figure 10 shows a Network-on-Chip with a 3 × 3 tile mesh topology and a typical router architecture from [45]. The router architecture presented has its input channels at the left and its output channels at the right. The switch fabric connects inputs with their respective output selected with the routing logic. But the arbitration logic controls which input channels use the switch fabric. RR is a typical arbitration policy to guarantee fair access; however, SuDO policy can control in a precise manner the bandwidth allocation to every input channel and avoid starvation. Several dataflows from diverse processor elements in different mesh network coordinates can converge in a link; therefore, they share the link’s bandwidth. SuDO can use the budget to control the share of bandwidth assigned to every flow. We can manage every link’s bandwidth share in every router in the pathway of a dataflow. This procedure will produce a smooth and more predictive behavior of the NoC-based system.

## 8. Conclusions and Future Work

In a scenario with multiple applications with dependent tasks where it is necessary to make a fine-grained control of the bandwidth allocation in a bus, it is better to use the SuDO policy since it is the one that has presented better results in the experiments carried out. While our arbitration policy guarantees bandwidth for each master if they do not need it—unlike the bandwidth waste of TDMA–, and opportunistically assigns the bus to the arbiter who needs it. To maintain fine-grained control of bandwidth allocation, SuDO has a debt monitor for each particular master. So both features: supervised debt and opportunistic sharing, give us a proper synergy to have fine-grained control of the bandwidth without wasting the bus utilization. However, the packet injection rate of the application causes that applications cannot reach the established weight ratio. It is necessary to adjust the bandwidth through the budget values to the packet injection rate of every master.

We have observed that one arbitration policy is more suitable than another depending on the type of scenario presented. For example, in a scenario with task-dependent applications where we do not desire to regulate the bus utilization, the policy that best fits is the Round-Robin policy because it is lightweight and avoids starvation and deadlock.

There are several aspects to follow as future work.

The ratio of support weights to the bus utilization percentage that a processing element tries to achieve is not straightforward. One of the reasons already mentioned is a limited packet injection rate of the application. This behavior suggests the possibility of coupling a regulator that monitors and dynamically adjusts in runtime the support weights about the request ratio of the processing elements.

On the other hand, there is a lack of studies where applications themselves generate heterogeneous packets. We believe that it is necessary to study and characterize this traffic type to fine-tune the bus utilization.

This work focuses on the behavior of the SuDO policy. The optimization of the hardware implementation in terms of maximum operating frequency, resource utilization, and energy consumption is desirable. The policy presented here can directly apply the effective bandwidth regulation of output ports in switches and routers. Furthermore, since the SuDO policy guarantees the differentiated allocation of bus bandwidth, it can simplify the mathematical analysis to measure SoCs, buses, switches, and Networks-on-Chip performance.

## Figures and Tables

**Table 1:** Related Work: Arbitration Policy & QoS Metrics

QoS	Round-Robin	Probabilistic	TDMA	Priority	Reconfig.	Others
Access	[25,26,27,28,29,30]	[31]				
Latency	[16,18,19,32,32]	[33]	[32]	[34,35]	[16,18,19,24,32,33]	
Utilization		[12,36,37]	[17,38,39]	[17]		[4,36,37,38,39]
EoS	[14]	[40]				[14]
Bandwidth	[2,15]	[41]	[2,17]	[15,17,21,22,23]		[8,20]

**Table 2:** Operational parameters and metrics used in evaluations.

Acronym	Name	Meaning
*P*	Politics	This parameter establishes the policy that will operate in the interconnection system 0—Round-Robin, 1—Weighted Round-Robin Modified [6], 2—SuDO (our proposal).
$W_{i}$	Weight	There is one entry for each master in the system, indicating the desired proportion of bandwidth the master must have. This parameter must have an integer value between 1000 and 10000 flits.
$U_{b}$	Bus Utilization	This variable accumulates the total number of clock cycles the bus is in use in a simulation.
$I_{b}$	Idle Bus	This variable accumulates the total number of clock cycles the bus is idle in a simulation.
$T_{appexec}\left(i\right)$	Application execution time	It is the time in clock cycles it takes an application(i) to execute all the tasks that compose it.
$T_{masterexec}\left(i,j\right)$	Master execution time	It is the time in clock cycles it takes the master(j) to transmit the flits generated by the tasks of the application(i)
$T_{xflits}\left(i,j\right)$	Transmited Flits time	Is the amount of flits that are transmitted by the master(j) when running the application(i) tasks.

**Table 3:** Origin of the implementation and meaning of the weight for each policy.

Policy	Origin	Associated Weight Meaning
RR	Williams and Towles [6]	The value associated with each of the masters has no meaning with this policy
LTY	Lahiri et al. [41]	Number of tickets assigned to each master
TDMA	Our implementation	Number of slots assigned to each master
WRR	Williams and Towles [6]	Weight in number of cycles for each master
WRRM	Williams and Towles [6]	Weight in number of cycles for each master
SuDO	Based on the architecture presented in this document	Budget in number of cycles for each master

**Table 4:** Summary of results achieved with the tests carried out. All three scenarios run with three task-dependent applications. Each of the applications mapped on eight processors, a total of 24 processors working in the system. The purpose of the tests is to observe the behavior of different arbitration policies behavior regarding their ability to control the bus bandwidth/utilization for the given weights.

Applications in the Scenario	Policies/Support Weights	Results
One FPPPP type application, two FFT-1024_complex type applications.	Policies: LTY, TDMA, and WRR. Weight ratio: (1/2/2) FPPPP = 1, FFT-1024 = 2	**LTY** fails to control the bus bandwidth utilization for each application. **TDMA** achieves bandwidth control at the cost of a notable reduction in performance. **WRR** generates a deadlock.
One FPPPP type application, two FFT-1024_complex type applications.	Policies: RR, WRRM, and SuDO Weights ratios: (1/1/1) FPPPP = 1, FFT-1024 = 1 (1/3/3) FPPPP = 1, FFT-1024 = 3 (1/2/2) FPPPP = 1, FFT-1024 = 2	**RR** does not control bus bandwidth utilization of each application. **WRRM** achieves coarse-grained bandwidth control, but produces a bias towards processors with larger packet sizes. **SuDO** achieves a fine-grained bandwidth control if an application have a low packet injection rate, others applications share the unused allocated bandwidth.
Three FFT-1024_complex type applications.	Policies: RR, WRRM, and SuDO (1,1,1) (1/3/3) (1/2/2).	**RR** does not control bus bandwidth utilization of each application. **WRRM** achieves coarse-grained bandwidth control, but produces a bias towards processors with larger packet sizes. **SuDO** achieves a fine-grained bandwidth control if an application have a low packet injection rate, others applications share the unused allocated bandwidth.

**Table 5:** Resources, operation frequency and power consumption for the hardware implementation of arbitration policies. * Denotes a different approach—a traffic regulator—for bandwidth control.

Policy	# Used Slice Registers	# Used LUTs	Max. Operating	Total On-Chip	Dynamic
	Available 106,400	Available 53,200	Frequency MHz	Power mW	Power mW
				Freq. 100 MHz	
Round-Robin	32–0.03%	66–0.12%	235.07	106	2
WRRM	280–0.26%	527–0.99%	190.11	115	10
SuDO	360–0.34%	1136–2.13%	80.38	114	10
ABE * [44]	582–0.54%	1131–2.12%	—	114	—
